# Unexpected recovery of COVID-19 related Pediatric acute liver failure in a patient with an immunosuppressive syndrome with conservative management

**DOI:** 10.1093/omcr/omaf246

**Published:** 2026-03-23

**Authors:** Ghada Abdulaziz, Kashif Majeed, Saleh Alshehri

**Affiliations:** Pediatric Critical Care Department, King Saud Medical City, Al Imam Turki Ibn Abdullah Ibn Mohammed Street, Ulaishah District, Riyadh 12746, Kingdom of Saudi Arabia; Pediatric Critical Care Department, King Saud Medical City, Al Imam Turki Ibn Abdullah Ibn Mohammed Street, Ulaishah District, Riyadh 12746, Kingdom of Saudi Arabia; Pediatric Critical Care Department, King Saud Medical City, Al Imam Turki Ibn Abdullah Ibn Mohammed Street, Ulaishah District, Riyadh 12746, Kingdom of Saudi Arabia

**Keywords:** Critical care medicine, paediatrics, Hepatology, Infectious diseases and tropical medicine, endocrinology and metabolism, Sanjad-Sakati Syndrome, Multi-inflammatory syndrome in childre

## Abstract

Background: We describe the first pediatric case of a 20-month-old female with Sanjad-Sakkati Syndrome who developed acute liver failure with associated multisystem inflammatory syndrome in children [МІS-C] and a good outcome. The primary finding in this case, is that despite the high morbidity and mortality rates of SSS patients due to its immunosuppressive effects, early aggressive approach to treating severe viral infections manifestations, for instance acute liver failure could lead to a favourable outcome and avoid the need for liver transplant.

## Introduction

The accepted definition of pediatric acute liver failure [PALF] includes both moderate coagulopathy [international normalized ratio [INR], > 1.5] with encephalopathy and severe coagulopathy [INR, > 2.0] without evidence of encephalopathy [1]. PALF could be a manifestation of severe acute COVID-19 infection, marking an excess in innate immunoinflammatory responses [2]. The Centers for Disease Control and Prevention [CDC] and World Health Organization [WHO] defined this condition as COVID-19-associated multisystem inflammatory syndrome in children [MIS-C]. Multisystem inflammatory syndrome in children [МІS-C] is a rare complication of COVID-19 that is characterized by prominent signs and symptoms of the gastrointestinal, cardiovascular, and mucocutaneous systems [2].

To date, there has been only one case report of SARS-COV2 infection with rapidly progressive fulminant liver failure in a previously healthy child, with a fatal outcome [3].

We present a case of fulminant acute liver failure in the context of MISC in an immunosuppressed patient who ultimately survived without the need for liver transplant.

### Case presentation

A 20-month-old female infant known case of Sanjad-Sakati Syndrome [SSS], presented to our hospital with 3 days history of persistent high-grade fever, cough, increasing respiratory effort, vomiting and watery diarrhea. Her family had been having upper respiratory tract infection symptoms for the past one week. The patient was on alfacalcidiol treatment for SSS and recently has been taking oral paracetamol for symptomatic relief. In the emergency department, the patient was having the dysmorphic phenotypic features of SSS, opening eyes to painful stimuli, severely dehydrated [markedly sunken eyes with tented skin turgor], cold extremities, delayed capillary refill time 4 seconds, rapidly breathing with no respiratory distress, hepatomegaly 4 cm below the costal margin and brisk reflexes. No stigmata of chronic liver disease were observed. The patient was febrile [Temperature 38.5 C] and tachycardic [Heart rate 130 beats per minute] but maintaining her blood pressure and oxygen saturation with room air.

**Figure 1:**
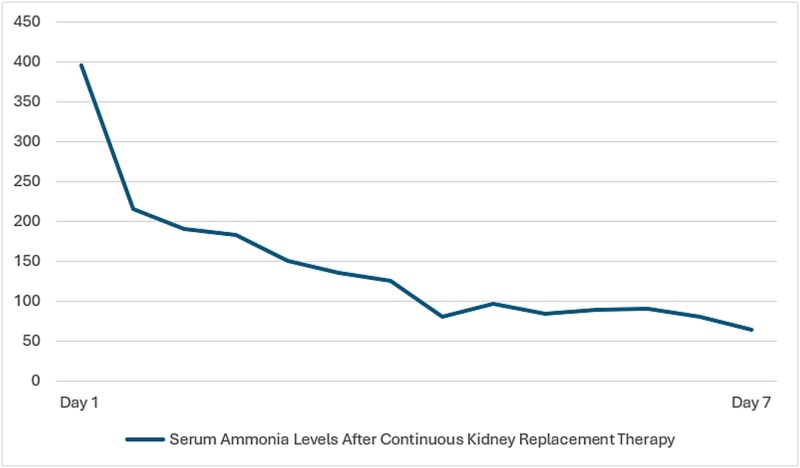
Continuous kidney replacement therapy for managing hyperammonemia.

SARS-CoV-2 PCR from nasopharyngeal swab was positive. Blood and urine cultures were negative. Laboratory workup was notable for thrombocytopenia, reduced absolute lymphocyte count and elevated absolute neutrophil count [Table 1]. Liver tests demonstrated acute liver failure [ALF] [Table 2]. Paraclinical tests were taken to clarify the underlying etiology of ALF by requesting hepatotropic viruses with negative results [Hepatitis A, B, C, Epstein–Barr virus, Cytomegalovirus, Herpes simplex virus types 1 & 2] and acetaminophen level was < 7 umol/L. Initial ammonia level was 345 umol/L [18–72 umol/L]. The high ammonia levels, perceived change in attention and brisk reflexes all pointed towards moderate to severe hepatic encephalopathy. On that basis, patient was admitted to PICU as a case of SARS-COVID 19 infection with acute liver failure and hepatic encephalopathy. Electroencephalogram [EEG] demonstrated moderate background abnormality with slowing confirming grade III hepatic encephalopathy. We restricted the intravenous fluid to 75% of maintenance fluids with Dextrose 10% one-half normal saline and supplemented with intravenous potassium chloride and potassium phosphate infusions. Our target serum glucose level ranged between 80–180 mg/dl. As for the hyperammonemia management, continuous renal replacement therapy was initiated from day 1 and continued for 7 days total, daily lactulose 0.5 cc/kg/dose was adjusted to produce minimum of four defecations per day and oral metronidazole. There was a rapid consistent drop in ammonia levels (Fig. 1). Due to the deranged coagulation profile, patient was transfused fresh frozen plasma and platelets before CRRT sessions. Patient was electively intubated due to her encephalopathy; head was elevated 30 degrees and serum Sodium levels were maintained between 145 and 155 mmol/L in fear of developing cerebral herniation with the high grade HE. We arranged for an urgent referral to liver transplant centre, however our case was rejected because of the poor long-term prognosis of SSS.

**Table 1:** Complete blood count upon admission to PICU. Blood workup is notable for thrombocytopenia, reduced absolute lymphocyte count and elevated absolute neutrophil count.

Complete blood count Parameters	Patient’s Values
White blood cells count	7.95 10^*^3/uL
Hemoglobin	8.45 g/dL
Hematocrit	27.90%
Mean Corpuscular Volume	99.30 fL
MCH	30.10 pg
MCHC	30.30 g/dl
Mean Platelet Volume	9.18 fL
Platelets	20.3 10^*^3/uL
Neutrophils %	63.60%
Lymphocytes %	27.60%
Neutrophils #	5.06 10^*^3/uL
Lymphocytes #	2.19 10^*^3/uL
Red Cell Distribution Width	20.70%

**Table 2:** Trend of liver enzymes values aaccording to the number of days of PICU admission.

Liver Function Test/Days of PICU Admission	Day 1	Day 2	Day 3	Day 4	Day 5	Day 6	Day 7	Day 8	Day 9	Day 10	Day 14
Alanine Aminotransferase (0–55) (U/L)	3899	7433	3881	3451	1119	1108	1223	736	610	490	125
Alkaline Phosphatase (156–369) (U/L)	165	181	247	342	369	393	314	357	304	247	170
Bilirubin, Total (5.1–20.5) (μmol/L)	48	103	161	253	254	260	216	289	227	270	124
Aspartate Aminotransferase (U/L)	26 413	HHH	4202	731	216	194	136	113	90	107	197

Due to the clinical severity of the case and confirmed COVID-19 infection, concerns for multisystem inflammatory syndrome in children MISC, and risk of progression to more florid hepatic involvement; patient was treated with intravenous immunoglobulin [IVIG] 2 g/kg/day and pulse steroids 30 mg/kg/day for 3 days. Additionally, the patient received remdesivir and methylprednisolone 2 mg/kg/day for 10 days total for the severe COVID-19 infection. Electrocardiography showed normal cardiac function. After 19 days of PICU admission patient was extubated and gradually weaned to room air. She returned to her baseline GCS 13–14/15. She graduated from the PICU to general paediatrics ward after 21 days. Fortunately, the liver recovered completely without the need for liver transplant.

## Discussion

Sanjad-Sakati syndrome [SSS] [OMIM 241410], also known as Richardson-Kirk syndrome or hypoparathyroidism-retardation-dysmorphism syndrome [HRDS], is a rare genetic disorder with autosomal recessive pattern of inheritance [4]. The syndrome was first reported from the Kingdom of Saudi Arabia by Sanjad et al in 1988 and has been confined to Arab Middle Eastern populations. It is a multisystem disorder characterized by severe intrauterine and postnatal growth failure, mental retardation, susceptibility to infections owing to low numbers of T-cell subsets and hyposplenisim, dentofacial anomalies and infantile-onset hypoparathyroidism that can result in hypocalcemic seizures. Mutation analysis postulated that SSS is caused by homozygous or compound heterozygous mutation in the Tubulin-specific chaperone E [TBCE] gene on chromosome 1q42, which is crucial for microtubule polymerization. Although some consider SSS as a variant from Kenny-Caffey syndrome type I [OMIM 244460] due to the shared biallelic mutation in the TBCE gene; they have been distinguished in the literature based on the skeletal phenotype and propensity to bacterial infections [4].

David et al, reported in a cohort of 63 patients with SSS and a mean age of 6.2 years, a total number of 775 hospitalizations mainly due to hypocalcemia [45%], fever [33%], seizures [25%], dehydration [13%], and hypoglycemia [12%]. In his study mortality rate was 52% [*n* = 33]. The median age of death was 2 years and 4 months with infectious diseases [*n* = 32] including pneumonia, septic shock, and meningitis being the commonest causes of mortality in that group of patients [5]. Similarly, Eli et al confirmed the high morbidity and mortality [55%] rates of this syndrome due mostly to infections [6].

Patients of SSS are liable to severe infections including recurrent pneumococcal infections, especially during the first 2 years of life [5, 6]. A recent clinical description of SSS patients by Odeya et al in 2023, classified SSS as a combined immunodeficiency syndrome [CID] with syndromic features [7]. Despite having normal B and T lymphocyte subsets, patients displayed impaired lymphocyte proliferation, poor T cell-dependent antibody responses and elevated IgA and IgE levels. Other features of immunodeficiency in Sanjad-Sakatti syndrome are hyposplenism and impaired neutrophil function [4].

Multiple studies on the outcome of SARS-COVID-19 infection in immunosuppressed pediatric patients have come to inconsistent conclusions [8, 9]. This has come to downsizing the detrimental effects of SARS-COVID-19 infection on immunocompromised children. Mayada et al explored the factors that predict severe COVID-19 infection in that group of patients. The study revealed that comorbid conditions [p = 0.025], absolute neutrophil count ≤ 500[p = 0.014] and absolute lymphocyte count ≤ 300 [p = 0.022] were predictors of severe viral infection [10].

The multiorgan involvement in COVID-19 infection has been attributed to the innate immune response triggering complement activation [11]. The pathologic immunodysregulation paves the way to dysregulated neutrophilia, macrophage activation, increased CD8 + T cells and B-cell plasma blasts and elevated interferon gamma. This hyperinflammatory Multisystem Inflammatory Syndrome in Children MIS-C has high ICU admission rates approximately 58% of cases;and manifests with persistent fever, cardiovascular dysfunction, severe gastrointestinal [GI], respiratory and neurological symptoms [12]. The clinical markers in the acute phase of MISC include elevated neutrophil and decreased lymphocyte and platelet counts, high serum C-reactive protein [CRP], ferritin, and D-dimers, and increased levels of proinflammatory cytokines [11]. Our patient upon admission had persistent high-grade fever for 3 days, multi-organ involvement including respiratory symptoms, fulminant acute liver failure and fluid responsive compensated hypovolemic shock. She also had positive COVID-19 PCR, low absolute lymphocyte count and platelets with elevated absolute neutrophil count, CRP and D-dimer levels, which confirmed the diagnosis of acute phase MIS-C. We opted to manage MIS-C by the administration of immunosuppressants [IVIG and methylprednisolone], in order to achieve the immune equilibrium and offset the inappropriate immune activation of severe COVID-19.

Liver involvement during SARS-COV 2 infection associated with MISC in the acute phase of infection has been described and is associated with worse respiratory outcomes in COVID-19 patients, requiring intensive respiratory support [50% vs 28%, p-value = 0.02] and prolonged duration of oxygen supplements [8 vs 5 days, p-value = 0.06] [13]. However, progression to acute liver failure is rare. Amanda et al in a retrospective cohort of 44 patients with MISC from US, reported that hepatitis was present in 19 subjects [43%] with only one progression to acute liver failure. She also reported that 21% of patients with hepatitis presented with shock compared to 0% in the non-hepatitis group, fortunately all had been discharged [14]. Chen et al from China had very similar results in a series of 43 patients with MISC and hepatitis, without any case of acute liver failure [15].

In the literature, there has been only one case report of SARS-COV2 infection with rapidly progressive fulminant liver failure and grade II encephalopathy in a 10-month-old previously healthy child. The patient was initially treated with IVIG, methylprednisolone and hyperhydration for the hyperammonaemia. Unfortunately, the case progressed to grade IV encephalopathy with refractory hyperammonaemia requiring haemodialysis without any improvement and ultimately died from refractory catecholamine resistant shock with DIC picture and multi-organ failure [3].

In our centre we tend to avoid over-hydration as it precipitates cerebral edema, ascites and pulmonary edema. On the contrary underhydration causes hepato-renal syndrome and worsening encephalopathy. Our patient presented with stage III hepatic encephalopathy confirmed by EEG and an ammonia level of 394, from the first day of admission we opted for a more aggressive approach by electively intubating the patient, initiating partial TBI protocol, continuous renal replacement therapy [CRRT] for 8 days total and lactulose 0.5 cc/kg/dose alongside appropriate hemodynamic support to ensure cerebral perfusion. Ammonia levels above 200 umol/L have been strongly associated with brain herniation and is the commonest terminal event related to hepatic encephalopathy. Early initiation of CRRT in patients with hyperammonaemia and grade III-IV encephalopathy in acute liver failure has been associated with rapid decline in ammonia levels and decreased mortality [16].

## Conclusion

To date, this is the first pediatric case report of fulminant acute liver failure in the context of MISC in an immunosuppressed patient. Prompt early aggressive therapy by CRRT, IVIG and pulse steroids, could ultimately lead to full hepatic recovery and avoid the need for liver transplant.
